# Design of Fuel‐Dependent, Complex‐Coacervate‐Based Synthetic Cells

**DOI:** 10.1002/chem.71099

**Published:** 2026-05-06

**Authors:** Anna‐Lena Holtmannspötter, Xiaoyao Chen, Yiyi Fu, Melisa Badem, Phillip S. Weiß, Nicolas Glück, Chase Kanitz, Veronika Rupp, Kaige Wang, Job Boekhoven

**Affiliations:** ^1^ Department of Bioscience School of Natural Sciences Technical University of Munich Garching Germany

**Keywords:** coacervates, fuel‐dependent synthetic cells, peptide design, peptides, synthetic biology

## Abstract

Fuel‐dependent synthetic cells are compartments that require fuel to emerge and sustain. Without fuel, they decay, serving as a selection pressure in fueling‐starvation experiments. Recently, we introduced fuel‐dependent synthetic cells based on droplets as a route to synthetic life. These droplets grow, divide, and decay under starvation conditions. However, which parameters influence their lifetime and their ability to divide remain unknown. Moreover, previous designs of these synthetic cells suffer from the accumulation of waste products of their chemical reactions. In this work, we offer design rules for chemically fueled complex coacervate droplets with a focus on hexapeptides. We find that incorporating tryptophan into peptide designs increases the peptides’ affinity for polyanions and thus their ability to form droplets. The resulting synthetic cells are longer‐lived and more waste‐resistant and can produce offspring when RNA is used as the polyanion. Placing the tryptophan close to the reactive C‐terminus further extends the droplet lifetime by slowing deactivation. Together, these results lead to a new series of peptides to produce waste‐resistant, RNA‐compatible, fuel‐dependent synthetic cells.

## Graphical abstract

Fuel‐dependent synthetic cells form and persist only in the presence of chemical fuel, decaying under starvation. We establish design rules for hexapeptide‐based coacervates, showing that tryptophan enhances polyanion binding, extends lifetime, and reduces waste sensitivity. Near the active site, tryptophan slows deactivation and enables division when RNA serves as the polyanion.

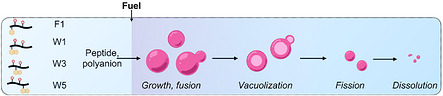

## 1 Introduction

**SCHEME 1:**
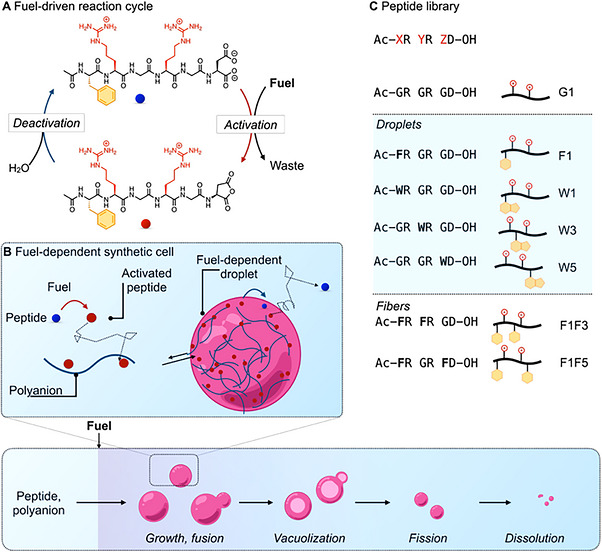
Modifying peptide designs to tune droplet behavior. (A). Peptide activation by fuel addition leads to a transient, droplet‐forming anhydride that hydrolyzes over time back to the peptide. (B). The activated peptide and our pSS polyanion form transient complex coacervate droplets. (C). Modifying the aromatic group and its positioning within the peptide sequence yields our peptide library.

Synthetic cells have been designed from the bottom up to emulate life‐like behaviors and offer insight into fundamental biology [1, 2, 3, 4, 5, 6, 7, 8, 9, 10]. For example, protocells have been developed in origin‐of‐life studies [11, 12] or proposed as a platform for de novo life [13], as compartmentalization is regarded as a critical requirement for life [14]. When combined with self‐replicating systems, such synthetic cells could become part of an ecosystem in which Darwinian evolution governs the ecosystem's fate [5, 15, 16]. In such Darwinian evolution experiments, selection pressure is an essential component, often captured through serial dilution experiments. As an alternative selection pressure, we recently introduced fuel‐dependent synthetic cells that require fuel for their “survival” [13, 17]. Like life, these synthetic cells are designed not to be stable at equilibrium, but instead to be maintained by a fuel‐driven reaction cycle [18]. The fuel‐driven activation step generates building blocks for the synthetic cells, whereas spontaneous deactivation returns the building blocks to their non‐assembling state. Consequently, the synthetic cell requires fuel to emerge and to grow, yet inevitably decays once activation can no longer outcompete deactivation. That interplay yields synthetic cells whose survival depends on fuel levels: when fuel is abundant, they nucleate and grow. When fuel is scarce, deactivation takes over, and compartments shrink as building blocks leave. Importantly, if fuel is supplied periodically, compartments can be rescued before they completely dissolve and, with repeated fuel pulses, can even grow larger. Periodic fueling thus creates a simple feast–famine ecology—compartments that last long enough to reach the next fueling cycle benefit from multiple growth opportunities, which imposes selection pressure on phenotypes that survive during starvation. Fuel‐dependent synthetic cells have been developed based on vesicles [19, 20, 21, 22, 23], oil droplets [24, 25], block copolymer micelles [26], DNA‐based droplets [27, 28], and complex coacervate droplets [29, 30, 31, 32, 33, 34, 35]. The complex coacervate‐based droplets are the focus of this work. These are based on a cationic peptide that complexes polyanions to form droplet‐based synthetic cells (Scheme 1A,B) [29]. The arginine‐containing hexapeptides react with carbodiimide fuel to generate an intramolecular anhydride at the peptide C‐terminus. This activation reaction increases the peptide's charge from neutral, zwitterionic to + 2, thereby increasing its affinity for charged polyanions and leading to droplet formation. Upon deactivation through hydrolysis, the peptide reverts to its original uncharged state, thereby weakening its interaction with the polyanion and leaving the dense phase. Thus, the addition of fuel to the system nucleates dynamic droplets that decay as fuel is depleted.

For these droplet‐based synthetic cells, many critical challenges still remain when using fuel‐driven droplets as synthetic‐cell components. First, the accumulation of waste progressively suppresses droplet formation. While inhibition of growth by waste is an inherent problem we also see in extant life, our fuel‐dependent synthetic cells are particularly sensitive to waste—already after one or two fueling cycles, inhibition of droplet growth is noticeable. Second, the peptides described in previous work do not form droplets with RNA. That is unfortunate, given RNA's central importance as an information‐bearing polymer in origin‐of‐life scenarios and as a substrate on which Darwinian evolution can act [36, 37]. Third, we recently revealed that fuel‐dependent droplets can produce offspring, a critical step toward the de novo synthesis of life [34]. However, the offspring was extremely short‐lived—usually, a droplet would break up into tens of tiny droplet fragments that only lived for a few tens of seconds. Thus, an ideal peptide for de novo life forms a fuel‐dependent droplet with RNA, is resistant to the waste produced after multiple fueling‐starvation cycles, and produces long‐lived offspring.

Here, we present a library of hexapeptides that form synthetic cells by complexing polyanions. We focused on the droplets’ resistance to waste, their ability to produce droplets with RNA, and their ability to produce offspring. We altered the hydrophobic and aromatic domains and their locations within the peptides, based on their importance in controlling phase separation in RNA‐binding proteins [38, 39, 40, 41, 42] and peptide‐based coacervate studies [43, 44, 45, 46, 47, 48]. We found that tryptophan was particularly favorable in the peptide to produce waste‐resistant droplets that produced offspring with RNA as a polyanion. We verified the ideal location for the tryptophan along the peptide backbone and eventually found three new droplet‐producing peptides, of which one was particularly waste‐resistant and could produce offspring producing droplets with RNA.

## 2 Results and Discussion

### 2.1 Peptide Design

We designed a library of hexa‐peptides based on our previously established peptide Ac‐FRGRGD‐OH (Scheme 1C, peptide F1). Peptide F1 is based on an acetylated N‐terminal phenylalanine (F), and two arginine glycine‐repeats (RG‐repeats) and a C‐terminal aspartic acid. The phenylalanine is the only hydrophobic and aromatic residue. The RG‐repeats are required to electrostatically bind polyanions. Finally, the C‐terminal aspartic acid is necessary for the chemical reaction cycle—it reacts with carbodiimides to be converted into its corresponding charge‐neutral anhydride. In peptide G1, we simplified this peptide and mutated the aromatic phenylalanine to glycine (Ac‐GRGRGD‐OH). Similarly, with peptide W1, we tested whether the more extended aromatic side chain of tryptophan (W) improved or worsened droplet behavior (Ac‐WRGRGD‐OH). We also compared the impact of tryptophan positioning at the N‐terminus (peptide W1, Ac‐WRGRGD‐OH), between the arginines (peptide W3, Ac‐GRWRGD‐OH), or near the C‐terminus (peptide W5, Ac‐GRGRWD‐OH). Finally, we further increased hydrophobicity by adding two peptides containing two phenylalanine residues at different positions (Ac‐FRFRGD‐OH, Ac‐FRGRFD‐OH, F1F3, F1F5). We reasoned that these designs are based on tryptophan, which has stronger cation‐π stacking and aromatic interactions with itself and aromatic polyanions than phenylalanine, due to a larger π‐electron system and indole moiety [45, 49, 50, 51, 52, 53]. Along these lines, previous work has shown that increasing the number of aromatic groups strengthens the interaction between polymers within coacervates [54]. For our comparison, we use polystyrene sulfonate as the polyanion, which we found in earlier work to easily make droplets with F1 [55]. We induced droplet formation by adding fuel and then observed the assemblies’ morphology, fuel‐starvation‐induced dissolution, and refuelability.

### 2.2 Design Dependent Synthetic Cell Morphologies

**FIGURE 1:**
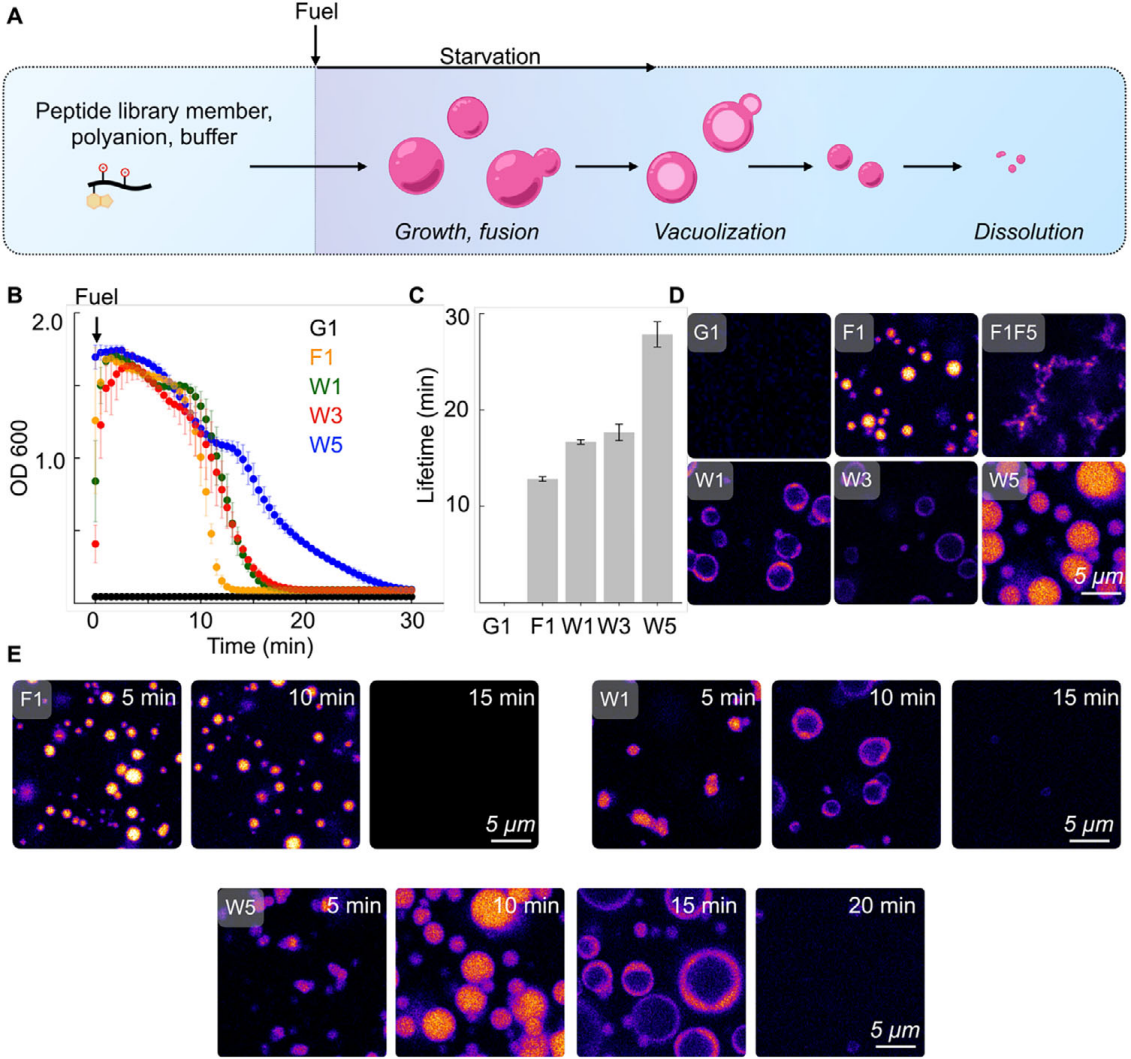
Aromatic residues control synthetic cell properties. (A). Schematic representation of the experiments: fuel is added to the peptide and polyanion in solution. (B). Turbidity traces for different peptides measured by a UV‐Vis spectrometer (12 mM peptide, 5 mM pSS, and 25 mM fuel). (C). Lifetimes of selected peptides derived from B. (D). Confocal micrographs of droplets formed by different peptides with pSS 10 min after fuel addition, stained with 500 nM Sulforhodamine. (E). Representative confocal micrographs over the course of the cycle for different peptides.

We used turbidimetry to monitor the assemblies' lifetimes with a spectrophotometer, tracking absorbance at 600 nm as a measure of scattering. (Figure 1B,C, and Table S2). We compared the peptides using 12 mM peptide, 5 mM polystyrenesulfonate (pSS), and 25 mM fuel. For all peptides except G1, turbidity developed in response to fuel. As G1 lacks any aromatic amino acids, we conclude that aromatic amino acids are critical for phase separation or self‐assembly. For all other peptides with one aromatic amino acid, that is, F1, W1, W3, and W5, turbidity emerged and decayed as fuel was depleted, typically within 30 min.

We defined the lifetime as the time at which the OD600 decreased below 0.12. The substitution of phenylalanine (F1) for tryptophan (W1) increased the lifetime from 13 to 17 min. Furthermore, between W1 and W3, we found no significant change in the lifetime. Surprisingly, changing the tryptophan location to W5 substantially increased the lifetime further to 28 min. These findings provide the first indication that the aromatic group is essential for the formation of active complex coacervates in our system—both its nature and its position in the peptide chain significantly affect the lifetime. The peptides containing multiple aromatic groups showed transient turbidity too (Figure S1). However, we observed an atypical, slowly increasing turbidity trace. Furthermore, we found that the solution became viscous, indicating more solid‐like assemblies and potential fiber growth.

To further examine the assemblies, we analyzed the samples using confocal microscopy. We found that 10 min after fuel addition, G1 showed no assemblies, F1 showed small droplets with radii less than one micron, and W1, W3, and W5 showed larger droplets with radii of a few microns (Figure 1D). Peptides containing multiple phenylalanines did not show any coacervate formation—for F1F5, we observed aggregates of incompletely fused droplets, which over time formed a fiber network that eventually dissolved (Figure S2A). Droplet fusion and fiber formation are consistent with the slowly increasing turbidity traces we observed. F1F3 showed similar behavior, but was overall more heterogeneous (Figure S2B). These findings indicate that multiple aromatic groups favor the formation of fibers and aggregates over droplets [56]. Therefore, the multi‐phenylalanine peptides were not further investigated.

We compared the morphologies of fuel‐dependent droplets throughout their reaction cycle. Peptide F1 formed small droplets that fused but never exceeded a few micrometers in diameter. These droplets eventually dissolved simply by shrinking after 10 min. The W‐containing peptides also formed droplets that grew by fusion in the first minute of the cycle. However, unlike F1, all the droplets formed by all W‐containing peptides grew larger and, as they dissolved, showed vacuolization. For W5, vacuoles were present for multiple minutes, during which vacuolized droplets could even fuse Sup. Movie (S1). At the end of the cycle, the vacuolized droplets shrank abruptly before dissolution. We found that F1 can form vacuoles and bigger droplets, but only when more fuel is added, and droplets thus grow larger (Figure S3). This finding is in line with our previous work: [57] droplets must exceed a threshold size to form fuel gradients within them, leading to vacuolization. From this work, it becomes clear that tryptophan‐containing peptides require less fuel to reach this size.

### 2.3 Tryptophan‐Based Synthetic Cells Show Better Refuelability

**FIGURE 2:**
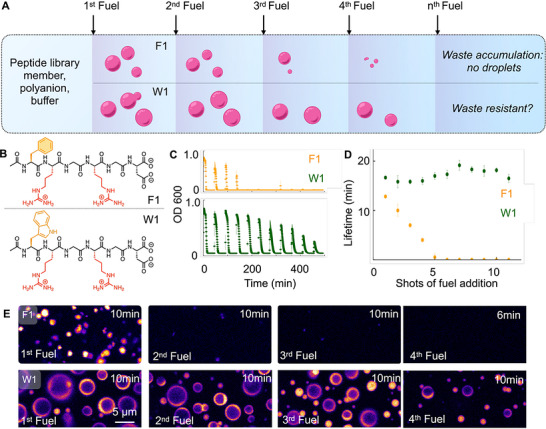
Tryptophan controls waste resistance. (A). Scheme of fueling and starvation experiments. (B). The sequences of peptides F1 and W1. (C). OD600 as a function of time when 25 mM of fuel is added every 45 min to 12 mM peptide, 5 mM pSS. (D). The lifetime of the droplets formed by F1 and W1 as determined from the data in C. (E). Confocal micrographs 10 min after the 1^st^, 2^nd,^ 3^rd^, and 4^th^ fuel addition.

We conclude that the W‐containing peptides form droplets that grow larger and live longer, likely due to stronger self‐interaction and interactions with the aromatic groups of the pSS. As these W‐containing peptides are more potent droplet‐formers than their phenylalanine counterparts, we set out to assess the droplets' ability to be refueled. For our synthetic life, it is critical that droplets that survive a starvation period and can grow with the next round of fuel (Figure 2A). However, refueling often leads to trapping or a complete lack of droplet formation due to the buildup of EDU as waste. We found that the droplets produced by peptide F1 suffered from the accumulated waste already after the second round of fueling with 25 mM EDC—the maximum achieved turbidity and droplets’ lifetime decreased (Figure 2B,C,D and E). With each additional round of fueling the system with 25 mM of EDC, the maximum turbidity and lifetime further decreased until, after 5 rounds, no turbidity was observed. We conclude that F1 is severely affected by EDU buildup. In contrast, the tryptophan‐containing peptide W1 could be refueled 10 times, at which point dilution becomes a more significant issue than waste buildup. The other tryptophan‐containing peptides also showed increased refuelability (Figure S4).

### 2.4 Tryptophan Location Affects Droplet Formation

**FIGURE 3:**
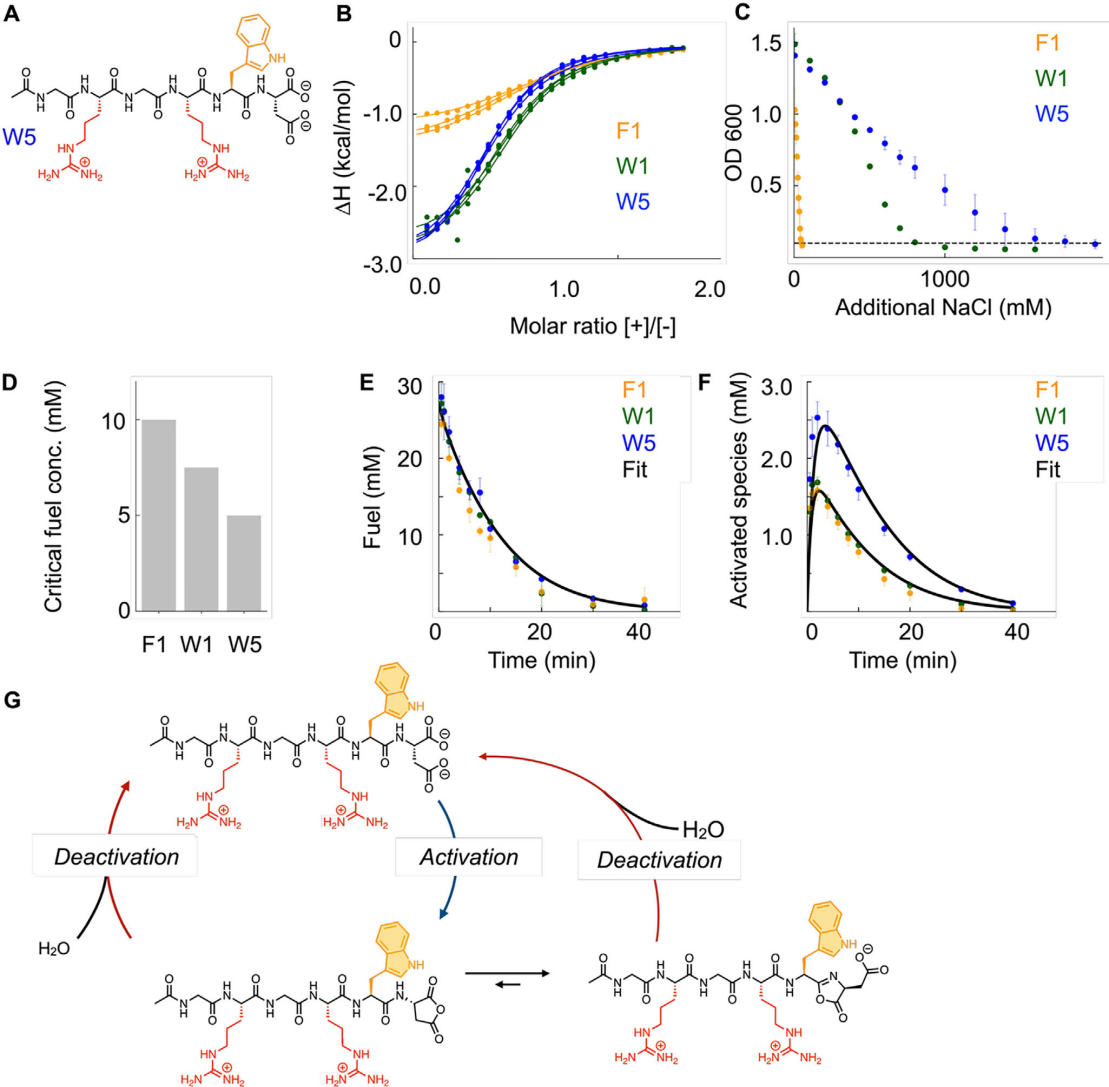
Tryptophan location affects droplet formation. (A). Chemical structure of peptide W5. Note the aromatic group position next to the active site. (B). ITC traces for different peptides with pSS. (C). The critical salt concentration for droplets formed with both peptides as measured by titrating static droplets with additional sodium chloride. (D). The critical fuel concentration to induce droplet formation under standard conditions. (E). EDC consumption under standard conditions as measured on HPLC and fitted with previously established kinetic model. (F). Anhydride concentrations after fuel addition as measured on HPLC and fitted with previously established kinetic model. (G). Proposed mechanism for activated species formation in W5.

From the data presented so far, it is clear that tryptophan in the peptide is better for producing fuel‐dependent droplets—the droplets live longer and are more waste‐resistant than those produced by phenylalanine‐bearing peptides. We hypothesize that the underlying mechanism involves increased binding strength between the peptide and the polyanion. To verify this mechanism, we tested the peptides' affinities for pSS using isothermal titration calorimetry (ITC; Figure 3A, 3B and Table S3). We observed approximately 2‐fold higher pSS‐binding enthalpies for W1 and W5 than for F1, yielding K_d_ values of 32 and 63 µM, respectively. This finding was further validated by measuring the critical salt concentration needed to dissolve droplets. In these experiments, we used a model peptide that does not hydrolyze. We prepared droplets with this peptide and titrated them with a NaCl solution. We observed a more than 10‐fold increase in the critical salt concentration from F1 to W1, indicating far stronger interactions with the tryptophan‐containing peptide (Figure 3C and Table S2). Along these lines, more salt was required to dissolve the W5 droplets than the W1 droplets. Similarly, we determined the minimal amount of fuel required to produce droplets—10 mM for F1, compared with only 7.5 mM for W1 (Figure 3D and Table S2). Surprisingly, this value was even lower for W5 at only 5 mM EDC. This is surprising given the similar binding affinities for W1 and W5.

The difference in critical fuel concentration, yet similar K_d_, prompted us to test the kinetics of the chemically fueled reaction cycle. As the C‐terminal aspartic acid is responsible for the activation reaction, we initially did not expect a drastic difference between the peptides. Indeed, when we monitored the fuel consumption, as a measure for the second‐order activation kinetics, we found no difference between any of the droplet‐forming peptides—F1, W1, and W5 all consume EDC with a second‐order rate constant of 0.0081 mM^−1^ min^−1^, which is typical for our reaction cycle (Figure 3E) [18, 24, 58, 59]. To determine the rate constant of deactivation of the peptides, we used a quenching method we recently introduced, which stops the activation by raising the pH and traps the anhydride by reacting it with an amine [60, 61]. Between F1 and W1, there was no difference in the deactivation kinetics, with both exhibiting a pseudo‐first‐order deactivation rate constant of 1.08 min^−1^ (Table S4). The kinetics of the entire cycle could be described with a kinetic model developed in earlier work [62]. From these data, we conclude that F1 and W1 follow the same kinetics of activation and deactivation. But, because of the tryptophan in W1 it has a higher affinity for polyanions, resulting in less fuel needed to make droplets, which are more salt‐ and waste‐resistant.

Surprisingly, W5 deactivated by hydrolysis much more slowly than both W1 and F1. Its deactivation could be described by an apparent rate constant of 0.59 min^−1^, corresponding to a half‐life of 70 s for the activated peptide. As a result, more activated peptides accumulate and persist for longer (Figure 3F). Besides, HPLC analysis revealed a new transient peak alongside the anhydride peak, suggesting a second transiently activated species (Figures S9,S10). We tentatively assign the slowed kinetics to a new equilibrium between the anhydride in the activated state and an oxazolone, facilitated by the neighboring indole group (Figure 3G, see Supporting Note 1). Combined with the greater affinity of W5 for the polyanion compared to F1, this explains the increased lifetime of the droplets and the lower critical fuel concentration.

### 2.5 Tryptophan Containing Peptide Enables the Formation of Peptide‐RNA Droplets

**FIGURE 4:**
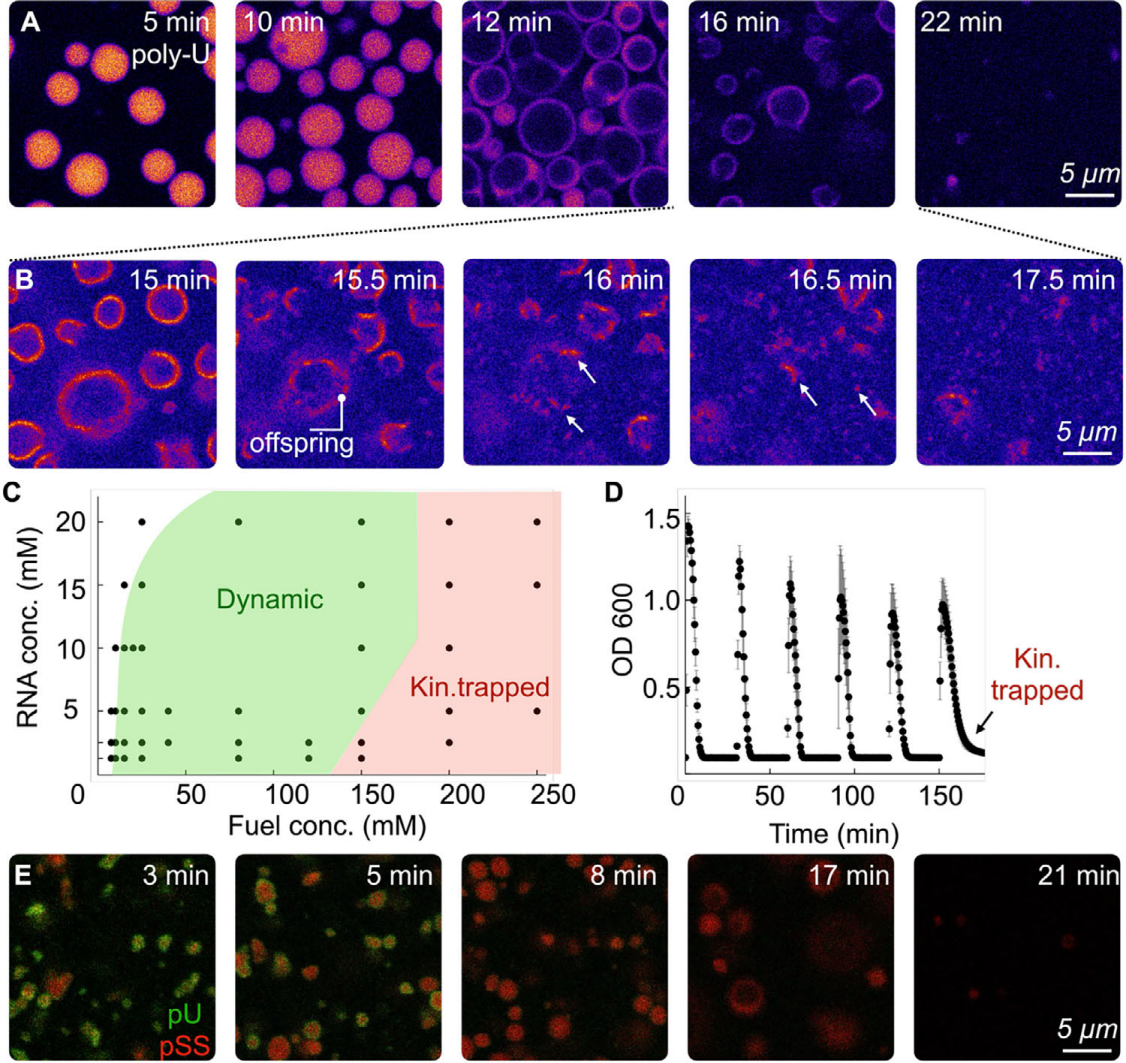
Tryptophan peptide enables the formation of peptide‐RNA droplets that produce long‐lived offspring fragments. (A). Confocal micrographs of peptide W5 with pU stained with Sulforhodamin B at selected points of the fuel cycle under standard polyU conditions. (B). Confocal micrographs during decay leading to offspring droplets that survive over a minute (highlighted with arrows). (C). Phase diagram of 20 mM W5 with different concentrations of pU and fuel. (D). OD600 as a function of time upon multiple fuel additions. (E). Confocal micrographs show colocalization of multiphase droplets of W5 (12 mM) with equal amounts of pSS and polyU (2.5 mM each) at selected points after the addition of 25 mM fuel. The droplets were stained with Cy3‐labeled pSS, and Cy5‐labeled A15‐ hybridized to the pU.

Using the better polyanion‐binding peptide that requires less fuel to form droplets, we tested polyanions other than pSS. To explore our fuel‐dependent synthetic cells as a platform for de novo life, we first tested RNA as a polyanion. The original peptide, F1, did not form droplets with RNA [18]. We hypothesized that the stronger aromatic interactions of the tryptophan, as well as the higher concentration of activated species, would make W5 a more suitable candidate. Notably, we found that coacervation with polyuracil (pU) was possible under various conditions, as well as phase separation with multiple other RNAs (Figures 4A and S5). For example, W5 could make droplets when we used the shorter, more structured tRNA and a long RNA produced via in vitro transcription with a high G‐content. In contrast, W1 formed only very small droplets with pU at very high fuel concentrations (Figure S6). We also returned to the peptides bearing multiple phenylalanines (F1F3, F1F5). Both were able to form transient droplets with pU, and, unlike pSS, we observed no fiber formation (Figure S6). Again, the droplets with F1F3 remained small even at high fuel concentrations, and both showed the formation of aggregates during droplet dissolution that remained for some time longer.

We focused on the more versatile W5 and poly U in greater detail and determined the K_d_ value for W5 to be 2.9 mM, while the K_d_ for F1 was not measurable (Figure S7 and Table S3). The lower affinity for this polyanion prompted us to choose a higher standard peptide concentration of 20 mM, keeping the polyanion concentration at 5 mM. With excess fuel (80 mM), we observed behavior similar to that of pSS, that is, the droplets grew by fusion, and large droplets formed vacuoles toward the end of the cycle (Figure 4A). Interestingly, the pU droplets that formed vacuoles broke apart non‐uniformly, leading to the production of offspring droplets that lived for several minutes (Figure 4B, Sup. Movie S2). In previous work, we attributed this behavior to the polydisperse nature of the polyanoin, in this case, pU. That polydispersity leads to the formation of solid speckles of long pU within the droplets that “survive” longer compared to the mother droplets. Notably, with our new W5 peptide, these fragments survive for up to several minutes Sup. Movie (S3), which is drastically longer than in our previous work. For our endeavours toward synthetic life, that is particularly appealing, as it gives us another selection pressure—the ability to produce long‐lived offspring. However, the weaker interactions with the polyanion limit the amount of fuel and waste that the system can tolerate. At 20 mM peptide, more than 150 mM fuel led to kinetic trapping of the droplets, that is, the droplets would not fully dissolve (Figure 4C). This could either be reached by one fuel addition or multiple smaller 25 mM fuel additions (Figures 4D and S8).

Finally, when we combined the peptide with both pU and pSS, we observed multiphase droplets in response to fuel—droplets with a core of pSS and a shell of pU. After 7 min, the pU shell was shed, leaving behind pSS droplets that finally also dissolved (Figure 4E). We conclude that it is possible to substitute an additional charge with a stronger hydrophobic group in a position that can slow down the hydrolysis of the active species. At the same time, the uncharged precursor species interacts less with the RNA, thereby preventing kinetic trapping of the droplets.

## 3 Conclusion

We investigated several hexapeptides and how their design affected the behavior of fuel‐dependent synthetic cells. These peptides are inspired by the disordered regions of proteins in biomolecular condensates, which are characterized by repeats of the RG motif. The necessity of a hydrophobic amino acid on top of these RG repeats became clear, as omitting it prevented droplet formation. Yet, more is not better—the presence of multiple aromatic groups in a single peptide led to the formation of transient aggregates and fibers instead of coacervates. The identity of the aromatic group strongly affected the ability to form droplets and the droplets' salt‐ or waste‐resistance. Substituting phenylalanine with tryptophan strengthened the interactions between the hexapeptide interactions, allowing for longer‐lived, larger droplets that could withstand more salt and be refueled more frequently. The position of the aromatic group in the peptide also proved important. When placed directly next to the activated aspartic acid, the tryptophan slowed down the hydrolysis of the activated species, leading to much longer‐lived droplets. Additionally, this enables the formation of synthetic cells with RNA, which, in turn, can lead to offspring production.

A central next step is to generalize these rules from pU to more sequence‐diverse, structured, and functional RNAs or RNA origami, and to quantify how RNA length, secondary structure, and charge density control droplet stability, vacuolization, and starvation‐induced offspring production. From a mechanistic perspective, the emergence of an additional transiently activated species in the tryptophan‐adjacent design motivates a broader exploration of activation chemistry (e.g., by stabilizing alternative activated states, such as thioesters) to extend compartment lifetimes without increasing fuel load or kinetic trapping. Functionally, longer‐lived offspring droplets open the door to coupling droplet “reproduction.” We envision refueling the solution precisely at the moment of offspring, and, thereby, “saving” the offspring to become the next generation. In that scenario, functional RNA that helps offspring production is directly inherited by the offspring. Ultimately, integrating RNA‐based informational polymers with sustained, waste‐resistant compartment cycling will enable experiments on genotype–phenotype coupling in purely chemical synthetic cells, moving these systems closer to Darwinian evolution.

## Conflicts of Interest

The authors declare no conflicts of interest.

## Supporting information



The authors have cited no additional references within the Supporting Information.

**Supporting File 2**: chem71099‐sup‐0002‐Movie_S1.avi

**Supporting File 3**: chem71099‐sup‐0003‐Movie_S2.avi

**Supporting File 4**: chem71099‐sup‐0004‐Movie_S3.mp4

## Data Availability

The data that support the findings of this study are available from the corresponding author upon reasonable request.
