# Supplementary material for: Design of Fuel‐Dependent, Complex‐Coacervate‐Based Synthetic Cells
Source: Chemistry. 2026 May 6;32(31):e71099. doi: 10.1002/chem.71099 (PMC13485242; doi:10.1002/chem.71099)
Supplement: Supplementary file 1 [file CHEM-32-e71099-s003.pdf]

Supporting Information for:

Design of Fuel-Dependent, Complex-Coacervate-based Synthetic Cells.

Authors: Anna-Lena Holtmannspötter,[1] Xiaoyao Chen,[1] Yiyi Fu,[1] Melisa Badem,[1] Phillip S. Weiß,[1] Nicolas Glück,[1] Chase Kanitz,[1] Veronika Rupp,[1] Kaige Wang,[1] Job Boekhoven [1]

Affiliations
[1] Department of Bioscience, School of Natural Sciences, Technical University of Munich, Lichtenbergstrasse 4, 85748 Garching, Germany

Materials and methods

We purchased 1-ethyl-3-(3-dimethylaminopropyl) carbodiimide (EDC), polyuridylic acid potassium salt (polyU), sodium chloride, sodium hydroxide,2-(*N*-morpholino)ethanesulfonic acid (MES) buffer, deuterium oxide, and Sulforhodamine from Sigma-Aldrich and used them without any further purification. General solvents were purchased from Sigma Aldrich in analytical or synthesis grade and used without further purification. Cy5-A15 was purchased from biomers.net GmbH. Amino acids and preloaded Wang resin were either ordered from Sigma Aldrich or Iris. Yeast tRNA was purchased from Invitrogen. PCR mastermixes and Purification kits for RNA and DNA were purchased from New England Biolabs. TBE buffer and sybr solution were ordered from Thermo Scientific. Agarose was ordered from Biozym.
Peptide F1 (AcFRGRGD-OH) was ordered from Caslo as the lyophilized TFA salt with a purity of >98%.All other peptides were prepared according to the method described below. Peptides were characterized before use with electrospray ionization mass spectrometry (ESI-MS) in positive mode and analytical HPLC as described in Supporting Table 1.

Peptide synthesis and purification. Ac-W(RG)$_2$D-OH (Ac-Trp-Arg-Gly-Arg-Gly-Asp-OH), Ac-G(RG)$_2$D-OH(Ac-Gly-Arg-Gly-Arg-Gly-Asp-OH), Ac-(GR)$_2$WD-OH (Ac-Gly-Arg-Gly-Arg-Trp-Asp-OH), Ac-GRWRGD-OH (Ac-Gly-Arg-Trp-Arg-Gly-Asp-OH), Ac-F(RG)$_2$FD-OH (Ac-Phe-Arg-Gly-Arg-Gly-Phe-Asp-OH), Ac-FRGFRGD-OH (Ac-Phe-Arg-Gly-Phe-Arg-Gly-Asp-OH) and Ac-W(RG)$_2$N-NH$_2$(Ac-Trp-Arg-Gly-Arg-Gly-Asn-NH$_2$) and Ac-(GR)$_2$WN-NH$_2$ (Ac-Gly-Arg-Gly-Arg-Trp-Asn-NH$_2$)were synthesized on a 0.5 mmol scale with a Liberty Blue® peptide synthesizer from CEM by standard Fmoc-SPPS. Pre-loaded Wang resin (Fmoc-Asp(OtBu) loaded, 100-200 mesh, 0.67 mmol/g) was used for the C-terminal acid peptides, Rink amide without preloading was used for the amidated version of the tryptophan peptides. A double Fmoc-deprotection and double coupling cycle with microwave heating was applied. Therefore, Fmoc-removal was performed twice per amino acid (2x 5 mL piperidine 20 v/v% solution in DMF, 90°C, 90 s) before each amino acid was coupled twice (2x 5 mL of 0.2 M amino acid in DMF, 2x 2 mL 0.5 M DIC in DMF, 2x 1 mL 1 M Oxyma pure in DMF, 90°C, 120 s each cycle). Arginine couplings were performed for 240 s at 90°C with the same reagent concentrations. Tryptophan, an amino acid, was used with side group Boc-

protection. Sidechain deprotection and final cleavage from the resin were achieved with a solution of 2.5% water, 2.5% triisopropylsilan, and 95% trifluoroacetic acid for two hours under continuous agitation at room temperature. Solvents were evaporated under a constant nitrogen flow, and following a precipitation with cold diethyl ether, the residue was dissolved in a mixture of water and acetonitrile. Further purification was done via preparative reversed-phase HPLC on an Agilent 1260 Infinity II setup (Agilent InfinityLab ZORBAX SB-C18 column (250 mm x 21.2 mm, 5 µm particle size). Separation was performed on an 18-minute linear gradient from 2 % to 75% acetonitrile in Milli-Q water, both with 0.1% TFA at a flow rate of 20 ml/min.

Sample preparation. 2.0 M stock solutions of EDC were freshly prepared by dissolving the EDC powder in MQ water. Stock solutions of polyU were freshly prepared by dissolving lyophilized polyU RNA in MQ water (41 mM, according to monomer units).
We prepared stock solutions of 500 mM MES buffer, the different peptides (50-75 mM), and the amidated peptides (30 mM) by dissolving the amount of the respective component in MQ water. Peptide and buffer stocks were adjusted to pH 5.3 by the addition of sodium hydroxide.
Samples were prepared by pipetting as follows: peptide, buffer, water, polyanion, EDC (fuel). Before and after fuel addition, the samples were mixed by pipetting. Standard conditions with pSS were 12 mM peptide, 5mM pSS, 200 mM MES buffer (pH 5.3), and 25 mM fuel. Standard conditions with polyU RNA were 20 mM peptide, 5mM polyU, 200 mM MES buffer (pH 5.3), and 80 mM EDC. Reaction cycles were started by the addition of the high-concentration EDC to the sample. We carried out all experiments at room temperature (RT). For more details, see the method described below.

ITC measurements. ITC titrations were performed using a MicroCal PEAQ-ITC instrument from Malvern Panalytical. The peptide concentration was kept constant at 2.5 mM (5mM charge), and the polyanion concentration was set to 500 µM for pSS and 1.1 mM for polyU (charge concentration). All experiments were performed at 25°C and with a control titration of the respective peptide concentration in 200 mM MES buffer (pH 5.3) into the same buffer without any polymer. All samples (cell and titration solutions) were prepared with the same concentration of MES buffer (200 mM at pH 5.3) to avoid additional dilution effects. The data were analyzed using a nonlinear least-squares algorithm implemented in the PEAQ-ITC analysis software. The component concentrations were selected in a regime in which no coacervation was observed.

Critical Salt concentration determination. We determined the critical salt concentration by titrating a 50 µL sample of static droplets. The conditions were 7 mM peptide, 5 mM amidated peptide, 5 mM pSS and 200 mM MES buffer (pH 5.3). We titrated with 1 or 5 M sodium chloride solution in 5 or 100 mM steps. Volumetric dilution was neglected because it was <25% at the maximum and was the same across all samples. After each addition, the sample was mixed by pipetting up and down, and then incubated for 30 seconds until air bubbles had disappeared. Afterwards, the

turbidity was measured at 25 °C. Dissolution was set as the turbidity falling below an absorption value of 0.1.

Critical Fuel concentration determination. We determined the critical fuel concentration by titrating a 50 µL sample with standard active droplet conditions with a 200 mM EDC solution in 2.5 mM steps. We defined the critical fuel concentration as the concentration at which turbidity exceeds 0.1.

Confocal Fluorescence Microscopy. We used a lightning SP8 confocal microscope (Leica) with a 63x water immersion objective to image the droplets. Excitation of Sulforhodamine was at 552 nm and detection with a HyD detector was from 565-735 nm. Excitation of the Cy5-tagged A_{15} RNA and the substrate and product oligomers was at 638 nm, and detection from 650-730 nm with a HyD detector. The laser intensities were set to 0.5% for PSS samples and 1 to 2% for polyU samples, increasing at the end of the cycle for better visibility of small fragments. The standard gain was set to 150 and the pinhole was set to 1 Airy unit.
Samples were prepared directly in microscopy well plate chambers (IBIDI, µ-slide Angiogenesis Glass Bottom) coated with a 5% PVA solution according to sample preparation described above. Samples were imaged on the glass to follow them over the course of the cycle. Micrograph images were contrast-adjusted and brightness-adjusted for better visibility.

Kinetic model. A kinetic model in python was used to fit the peptide anhydride, EDC and precursor concentrations based on HPLC analysis. This model has been described in detail in our previous work.[1, 2, 3] The rate constants used are described in Supporting Table 4. The kinetic model is based on the following reactions:

Reaction 0: Direct hydrolysis of the carbodiimide fuel to the urea waste product in the absence of peptide. The first order rate constant was obtained by fitting the EDC consumption in 200MM MES at pH 5.3. The value was determined to be 0.0045 min^{-1}.

Reaction 1: Formation of the O-acylisourea intermediate by the reaction of the C-terminal aspartic acid with the carbodiimide fuel. The rate constant was determined by fitting the EDC consumption in the presence of peptide in HPLC analysis. Its value was determined to be 0.0081 $mM^{-1}min^{-1}$.

Reaction 2: Formation of the anhydride from the O-acylisourea. The shortlived O-acylisourea cannot be observed by HPLC analysis and the rate constant can not be experimentally determined. It is derived from fitting the data.

Reaction 3: Hydrolysis of the O-acylisourea to the acid, an unwanted side reaction. It can also not be measured directly. The ratio of k3/k2 is given in the rate constants as an estimation of the degree of side reaction and derived from the fitting.

Reaction 4: Hydrolysis of the anhydride product to the acid. It is derived from the HPLC experiments with the pentylamine quench method and fitted with the (pseudo)-first order rate constant k_4=0.59 min^{-1} for W5 and k_4=1.08 min^{-1} for F1 and W1.

Analytical HPLC. Analytical reversed-phase HPLC experiments were performed on a ThermoFisher Vanquish Dual System. A 21 minute linear gradient from 2% to 60% ACN and MilliQ water, both with 0.1% TFA, was used for analyte separation on a ThermoScientific Hypersil GOLD column (250 x 4.6 mm, 5 µm particlesize). The standard detection wavelengths were set to 220 and 254 nm. The data was collected and analyzed with the Chromeleon 7 Chromatography Data System Software. For quantification of the peptide anhydrides during the reaction cycle, a quenching methodwith pentylamine was used.7 Specifically, after the reaction cycle was started by the addition of EDC stock, 20 µL of the corresponding sample were pipetted on 20 µL of a 400 mM pentylamine solution in acetonitrile inside of a 200 µL HPLC vial inlet and mixed by pipetting and vortexing. The turbidity vanished completely during the quenching and mixing process. Additionally, the pH of the quenched mixtures increased instantly which also inhibits further hydrolysis of the remaining EDC during the analysis and stops the reaction cycle. Separation was achieved by applying the abovementioned standard method for analytical HPLC experiments. The peptide anhydride concentrations were quantified from the corresponding peptide-pentylamine peaks using the same calibration curve for the signal at 254nm.

Turbidity measurements. The Turbidity measurements were carried out at 25°C using a Multiskan FC (ThermoFisher) microplate reader. Samples (50 µL) were directly prepared into a 96 well-plate (tissue culture plate non-treated). Every 30 seconds the absorbance of the samples was measured at 600 nm. Experiments were performed in triplicate.

RNA synthesis. RNA was synthesized via in vitro transcription based on DNA template sequences generated on NUPACK using specific constraints related to G-content (e.g. 50% G in this case). The DNA templates were amplified according to the manual of the NEB Phusion® High-Fidelity PCR Master Mix with HF Buffer (NEB #M0531) kit using 50µL reaction volume, 2.5 ng DNA template and 1 µL DMSO. The thermocycling conditions were chosen according to the manual with an annealing temperature of 68°C. The DNA was purified according to the NEB Monarch Spin PCR and DNA clean up kit (NEB #T1130). The concentration was measured using a Nanodrop spectrophotometer. The purity was assessed by running a 2.5% agarose gel in 1x TBE stained with Gel Red, for 90 minutes at 100 V. RNA was amplified according to the manual of the NEB HiScribe® T7 High-yield RNA synthesis kit (NEB #E2040) with a 50 µL reaction volume and a three-hour incubation time. The amplified RNA was treated with 2 µL of DNase I and incubated at 37°C for 15 minutes. 500 mM EDTA was added to reach a final concentration of 100 mM EDTA. The RNA cleanup was performed according to the NEB Monarch Spin RNA cleanup kit (NEB #T2050). The concentration was measured using a Nanodrop spectrophotometer. The purity was assessed by running a 10% Acrylamide/Bis gel (made with 10 µL TEMED and 100 µL APS) for 120 minutes at 150V. The gel was stained in a 0.01% SYBR Gold solution in TBE for 10 minutes.

Supporting Tables

Supporting Table S1: Characterization of peptides.

Name	Amino acid sequence	Mass calculated [g/mol]	Mass observed [g/mol]	Retention time* [min] at 254nm
F1	Ac-F(RG)$_2$D-OH	Mw=748.80	$[HMw]^+$ 749.48<br>$[H_2Mw]^{2+}$ 375.49	10.43
W1	Ac-W(RG)$_2$D-OH	Mw=787.84	$[HMw]^+$ 788.49<br>$[H_2Mw]^{2+\cdot}$ 395.01	10.97
G1	Ac-G(RG)$_2$D-OH	Mw=658.67	$[HMw]^+$ 659.49<br>$[H_2Mw]^{2+\cdot}$ 330.43	6.70
W3	Ac-GRWRGD-OH	Mw=787.84	$[HMw]^+$ 788.59<br>$[H_2Mw]^{2+\cdot}$ 395.06	10.50
W5	Ac-(GR)$_2$WD-OH	Mw=787.84	$[HMw]^+$ 788.58<br>$[H_2Mw]^{2+\cdot}$ 395.03	10.89
F1F5	Ac-FRGRFD-OH	Mw=838.92	$[HMw]^+$ 839.55<br>$[H_2Mw]^{2+\cdot}$ 420.58	13.43
F1F3	Ac-FRFRGD-OH	Mw=838.92	$[HMw]^+$ 839.61<br>$[H_2Mw]^{2+\cdot}$ 420.64	13.03
F1-NH2	Ac-F(RG)$_2$N-NH$_2$	Mw=746.83	$[HMw]^+$ 747.47<br>$[H_2Mw]^{2+}$ 374.52	10.02
W1-NH2	Ac-W(RG)$_2$N-NH$_2$	Mw=785.87	$[HMw]^+$ 786.50<br>$[H_2Mw]^{2+\cdot}$ 394.06<br>$[TFAMw]^+$ 899.73	10.58
W5-NH2	Ac-(GR)$_2$WN-NH$_2$	Mw=785.87	$[HMw]^+$ 786.48<br>$[H_2Mw]^{2+\cdot}$ 394.04<br>$[TFAMw]^+$ 899.75	10.09

*Gradient conditions: 21 min gradient 2%-60% ACN, total runtime 30 min

Supporting Table S2: Critical fuel and salt concentrations and lifetimes for different peptides with pSS under standard conditions.

Name	Amino acid sequence	Critical salt conc.	Critical fuel conc.	Lifetimes
F1	Ac-F(RG)$_2$D-OH	42 (+/-6)	10 mM	12.8 (+/- 0.24)
W1	Ac-W(RG)$_2$D-OH	833 (+/-125)	7.5 mM	16.7 (+/- 0.24)
W5	Ac-(GR)$_2$WD-OH	1733 (+/-339)	5.0 mM	27.8 (+/- 1.31)
W3	Ac-GRWRGD-OH	-	-	17.7 (+/- 0.85)

F1F3	Ac-FRFRGD-OH	-	-	19.0 (+/- 0.71)
F1F5	Ac-FRGRFD-OH	-	-	39.2 (+/- 0.62)

Supporting Table S3: Dissociation constants (Kd) and binding enthalpy for different peptides with pSS and polyU measured on ITC.

Name	Amino acid sequence	K_d (pSS)	K_d (polyU)	ΔH (pSS)	ΔH (polyU)
F1	Ac-F(RG)$_2$D-OH	62.9 µM (+/- 2.2)	x	-1.45 (+/- 0.14)	x
W1	Ac-W(RG)$_2$D-OH	32.3 µM (+/- 1.7)	2.93 mM (+/- 0.25)	-3.08 (+/- 0.12)	-1.32 (+/-0.16)
W5	Ac-(GR)$_2$WD-OH	32.4 µM (+/- 2.9)	2.93 mM (+/- 0.19)	-3.21 (+/- 0.05)	-1.19 (+/-0.12)

Supporting Table S4: Rate constants for the kinetic model. Ac=precursor peptide, An=anhydride, O=O-acylisourea.

Name	K0 (EDC→EDU)	K1 (Ac+EDC→O)	K3/K2 K2:(O→An+EDU) K3:(O→Ac+EDU)	K4 (An→Ac)
F1	0.0045	0.0081	0.000001	1.08
W1	0.0045	0.0081	0.000001	1.08
W5	0.0045	0.0081	0.000001	0.59

Supporting note 1:

We tentatively hypothesize the oxazolone formation as the most likely underlying mechanism of slower hydrolysis and higher accumulation of activated species for W5, based on several indications: 1. We observe the formation of a second transient species in the HPLC when injecting the peptide after fuel addition without the quenching (Fig. S9). When applying the quench method for F1 and W1, we observe only one double peak, corresponding to the different regioisomers of the pentylamine opening the anhydride at the two different carboxylic acids. Upon quenching W5, we observed a second double peak with a higher retention time (Fig. S10). This peak is transient, just as the quenched anhydride. Moreover, ESI mass spectrometry showed all four peaks have the same mass as the amide of the quenched anhydride. As the peaks have the same mass but different retention times, we suspect this is due to epimerization. This loss of stereo information could be explained by the formation of a cyclic species, most likely the oxazolone. We postulate that the anhydride and the

oxazolone are in equilibrium, with the oxazolone being stabilized by the indole moiety as well as the hydrophobic nature of the tryptophan. Tryptophan is known to interact strongly with carboxylic acids close to their indole moiety, for example, at membrane interfaces. Definitive proof of the oxazolone species or epimerization via ^1H and 2D NMR has proven challenging, due to the short-lived nature of the oxazolone and the overlapping of the MES buffer and EDC peaks with the aspartic acid CH_2 protons and the chiral proton being very close to the water suppression. Measuring NMR kinetics (without buffer), we see a non-transient second peak for the aspartic acid CH_2 protons appearing (Fig. S11).

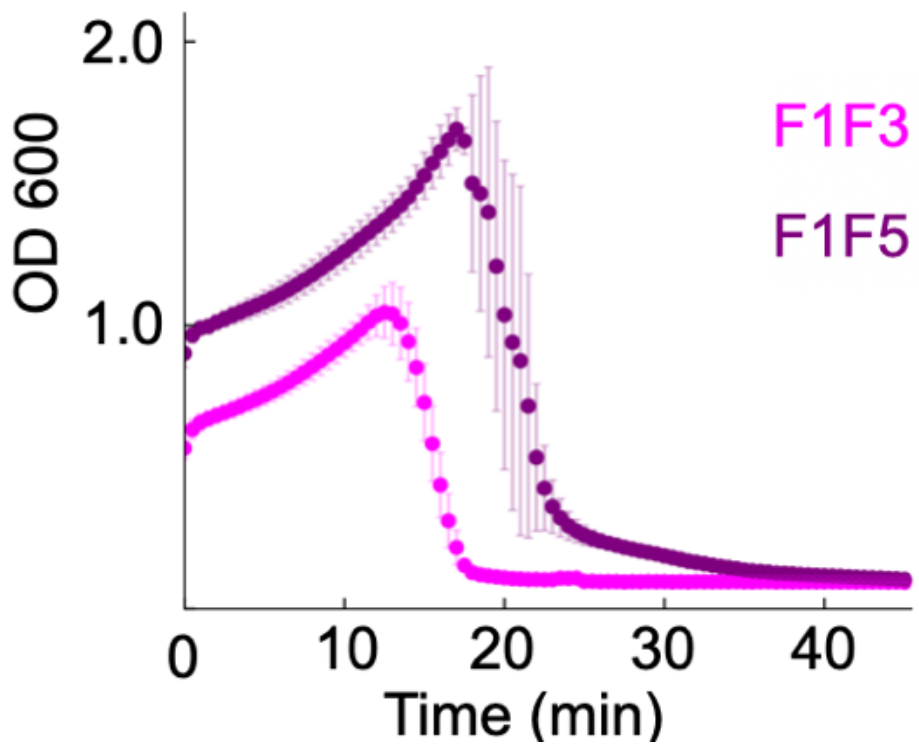

Fig. S1. Turbidity traces of F1F5 with polystyrene sulfonate under standard conditions. *Droplets were prepared according to standard active droplet sample preparation with 25 mM of fuel. Turbidity was observed as Absorbance at 600 nm on the UV-VIS spectrometer for forty-five minutes. Error bars represent the standard deviation from the average of 3 measurements.*

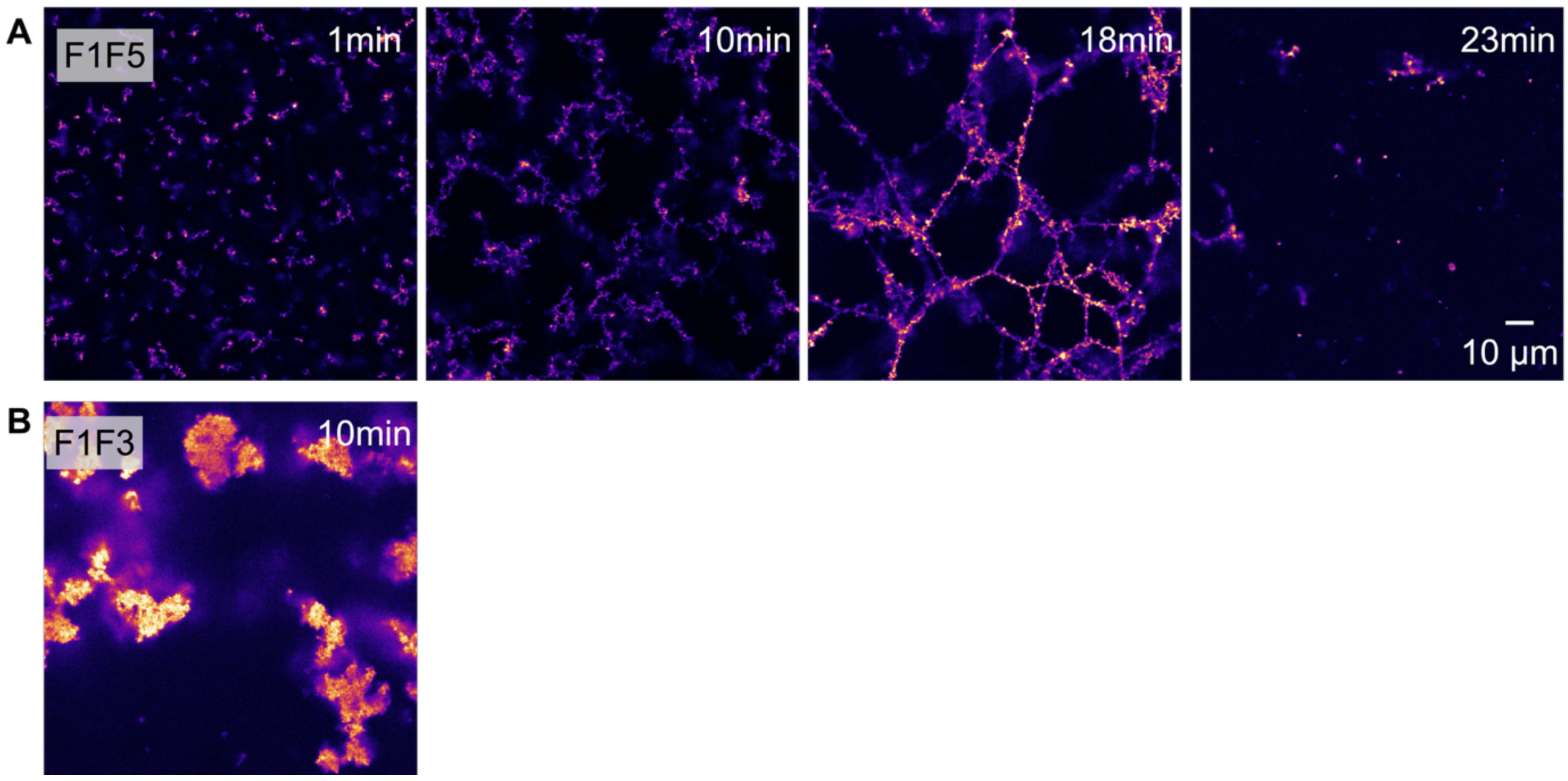

Fig. S2. Confocal micrographs of F1F3 and F1F5 at selected points of the cycle. *Samples were prepared according to standard active droplet conditions and stained with 500 nM Sulforhodamine.*

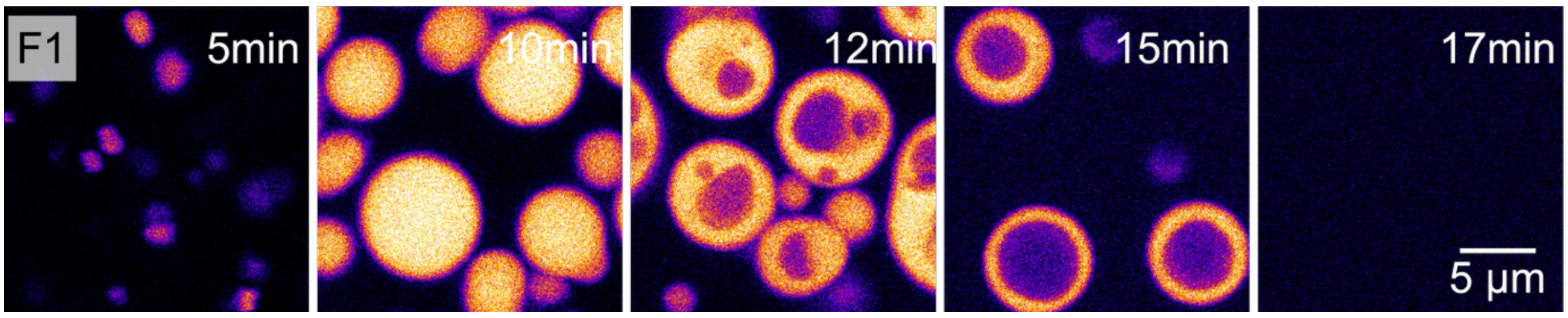

Fig. S3. Confocal micrograph of F1 at selected timepoints under higher fuel-droplet conditions. *Samples were prepared with 12.5 mM peptide, 12.5 mM PSS and 50 mM fuel and stained with 500nM Sulforhodamine. We observe the droplets for the duration of the cycle on the confocal.*

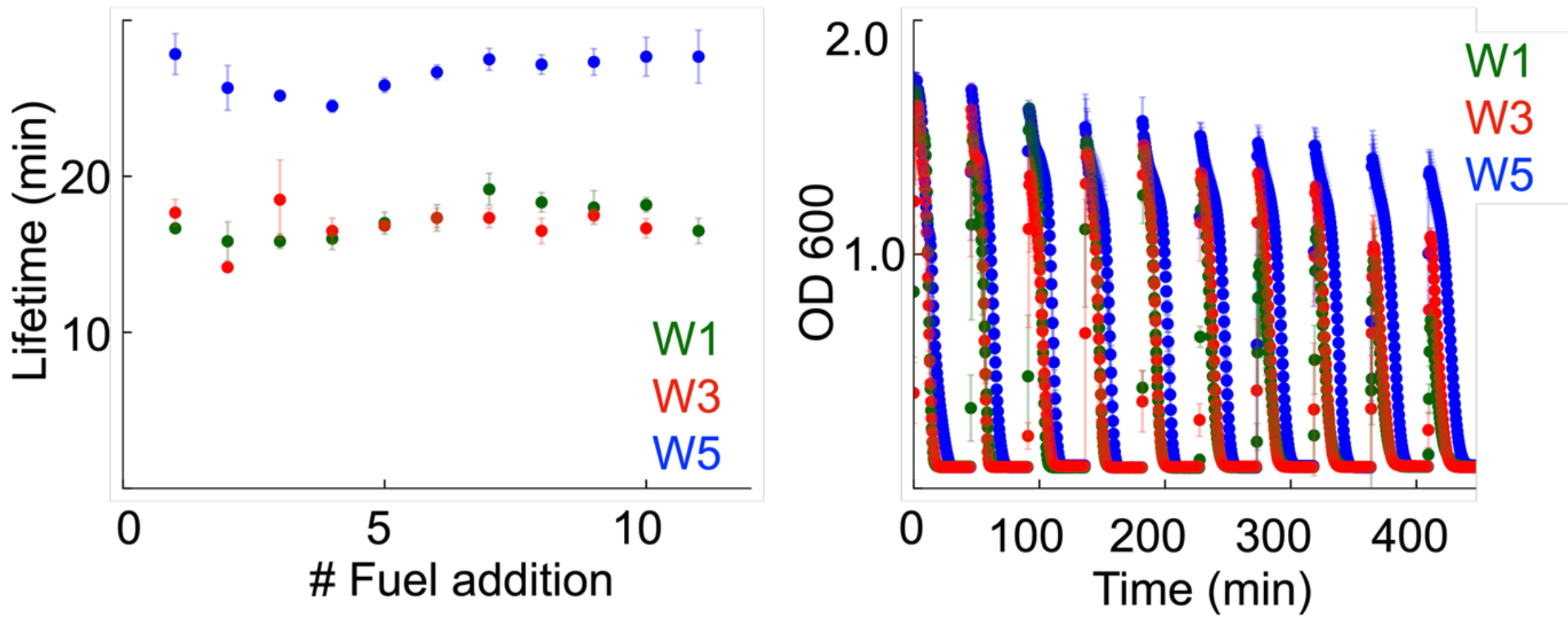

Fig. S4. Lifetime shift after multiple manual fuel additions for different tryptophan-containing peptides. *Droplets were prepared according to standard active droplet sample preparation with 25 mM of fuel. Turbidity was observed as Absorbance at 600 nm on the UV-VIS spectrometer for forty-five minutes after each fuel addition. Error bars represent the standard deviation from the average of 3 measurements.*

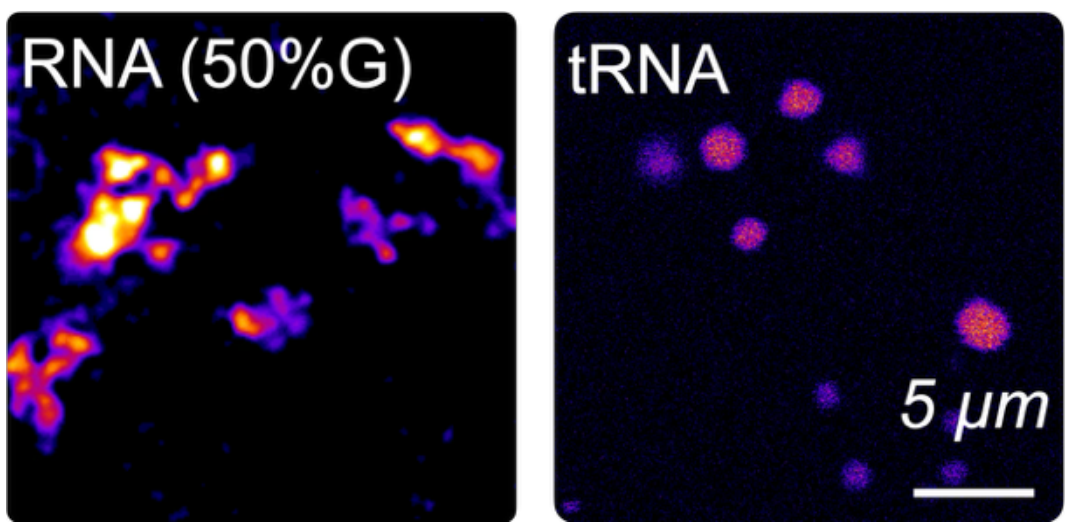

Fig. S5. Confocal micrograph of W5 with selected RNAs. *Samples were prepared with 20 mM W5 peptide, 5mM polyU and fueled with 25 mM EDC, staining with 1μM Sulforhodamine. The first micrograph has been treated with a Gaussian blur filter for clarity and better visibility.*

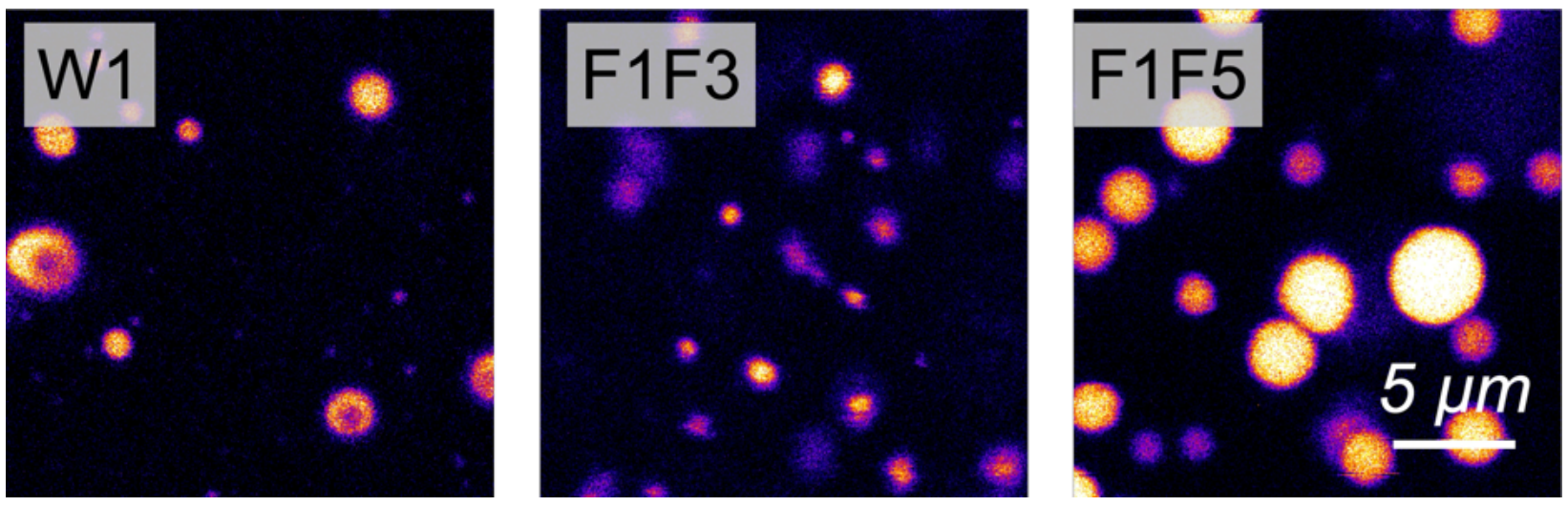

Fig. S6. Confocal micrograph of W1, F1F3, and F1F5 with polyU. *Samples were prepared with 20 mM W5 peptide, 5mM polyU, and fueled with 25 mM EDC, staining with 1µM Sulforhodamine. The first micrograph has been applied to a Gaussian blur filter for clarity and improved visibility.*

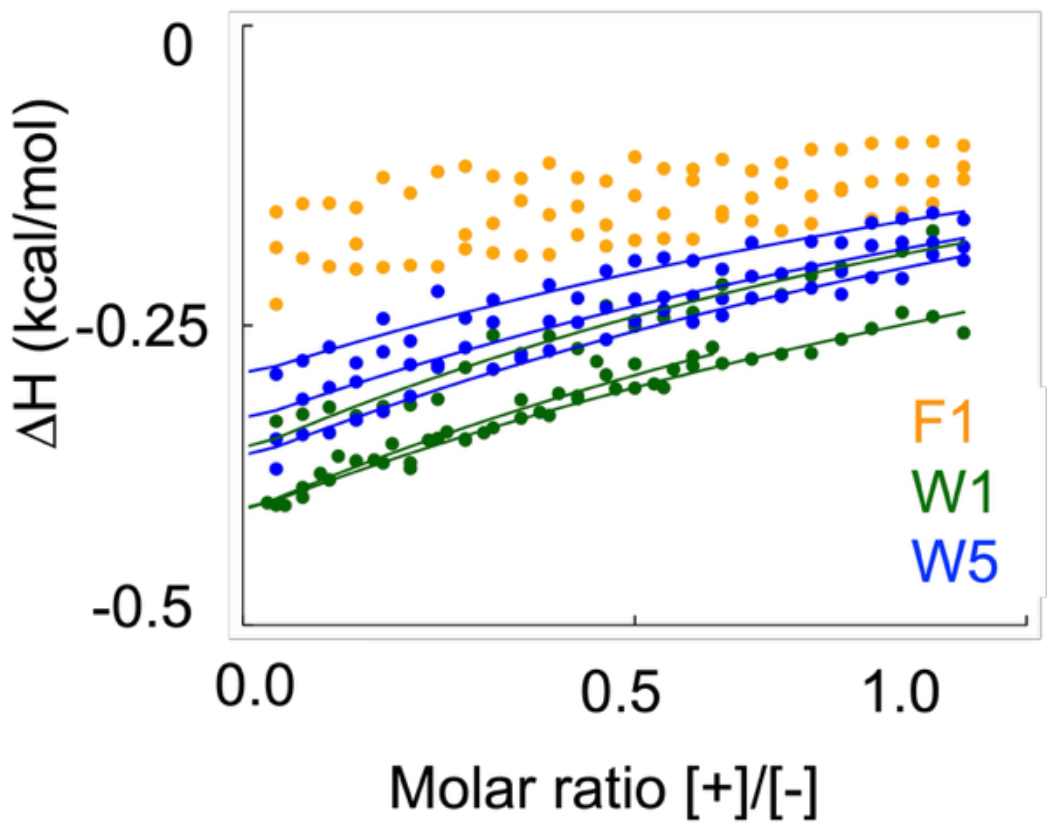

Fig. S7. ITC traces of different peptides with poly U RNA

Samples were prepared according to the method described above, peptides were used at 2.5 mM and RNA at 1.1mM (monomer concentration), both dissolved in 200mM MES (pH 5.3).

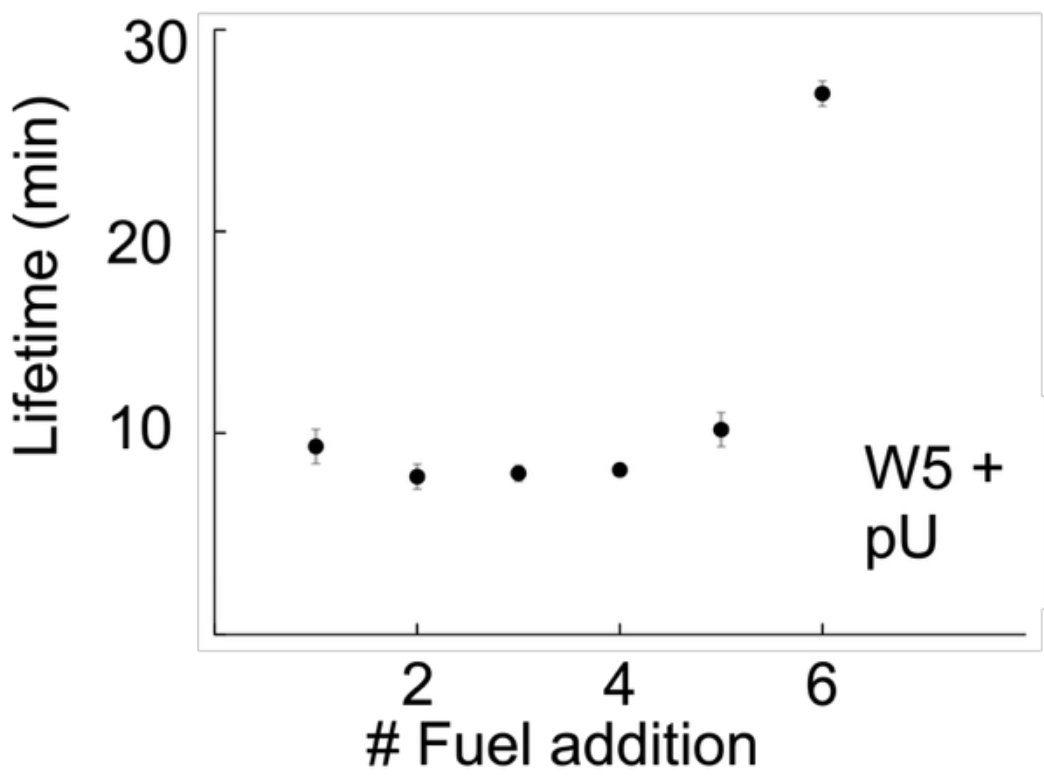

Fig. S8. Lifetime upon refueling for W5 peptide with polyU. Samples were prepared with 20 mM W5 peptide, 5 mM polyU RNA and 25 mM EDC in 200 mM MES buffer (pH 5.3). Fuel was added every 30 minutes and the lifetime defined at a threshold of 0.12.

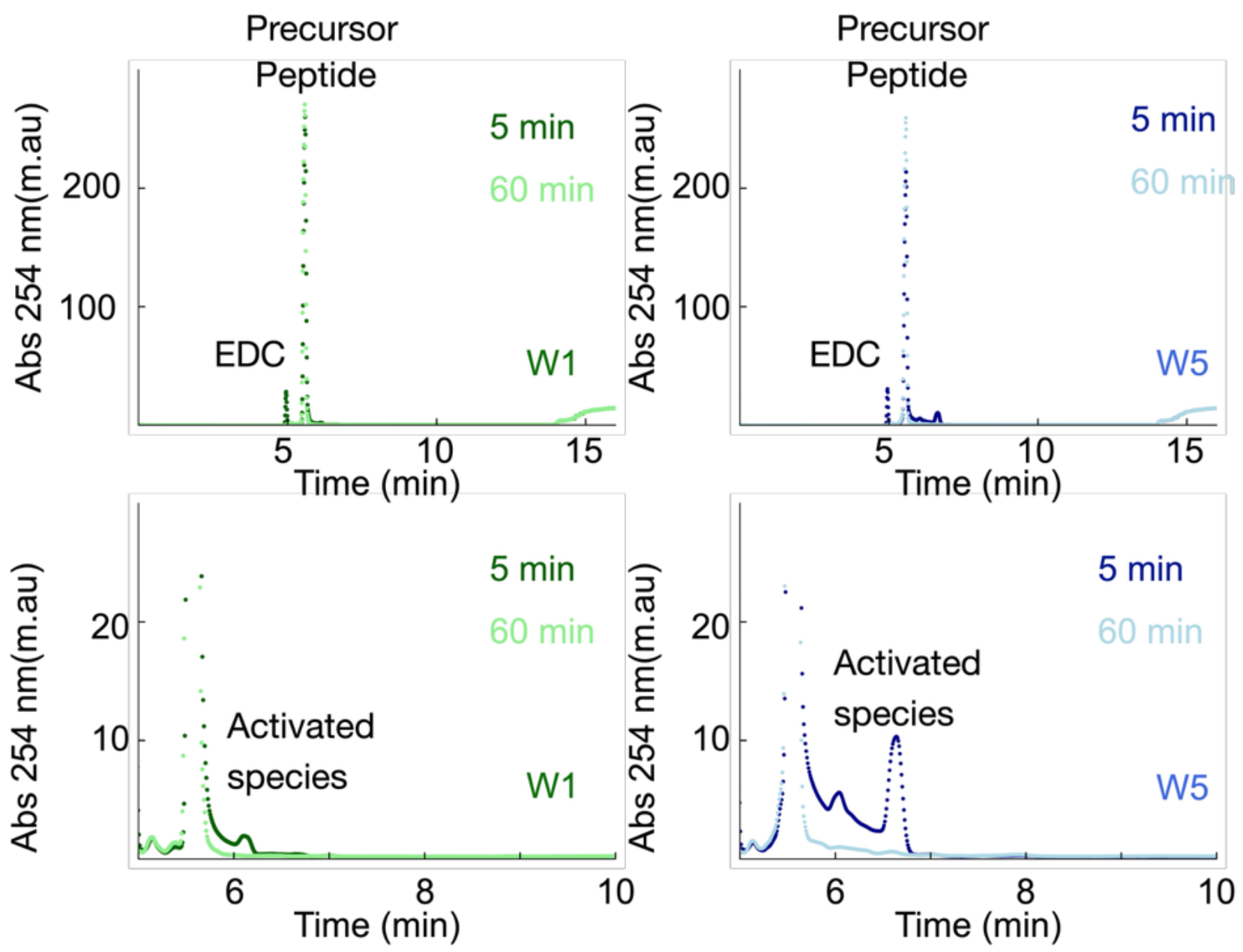

Fig. S9. Raw HPLC traces of unquenched kinetics for W1(green) and W5 (blue). Upper panels are the whole chromatogram, lower panels zoomed in on the activated species. Samples were prepared with 10 mM W5 or W1 peptide and 100 mM EDC in 200 mM MES buffer (pH 5.3). Samples were injected directly after fuel addition and after one hour. An isocratic HPLC method using 25% acetonitrile was selected for rapid separation.

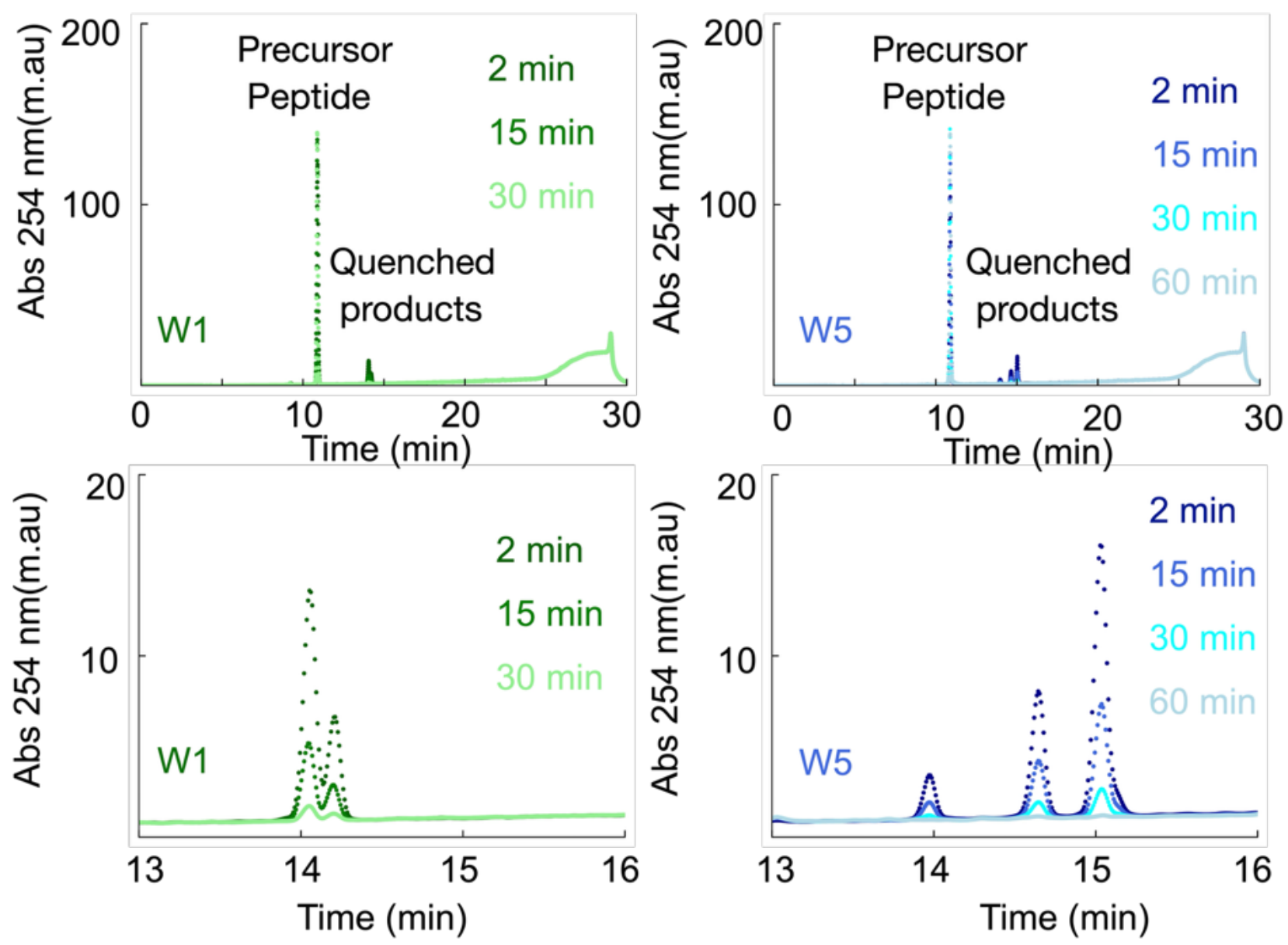

Fig. S10. Raw HPLC traces of quenched kinetics for W1 and W5. The upper panels show the entire chromatogram; the lower panels are zoomed in on the activated species. Samples were prepared with 12 mM W5 or W1 peptide and 25 mM EDC in 200 mM MES buffer (pH 5.3). Aliquots were taken at selected timepoints after fuel addition and immediately quenched 1:1 with a 400 mM solution of pentylamine in acetonitrile. Samples were injected (1μL) into the HPLC after 24 h incubation.

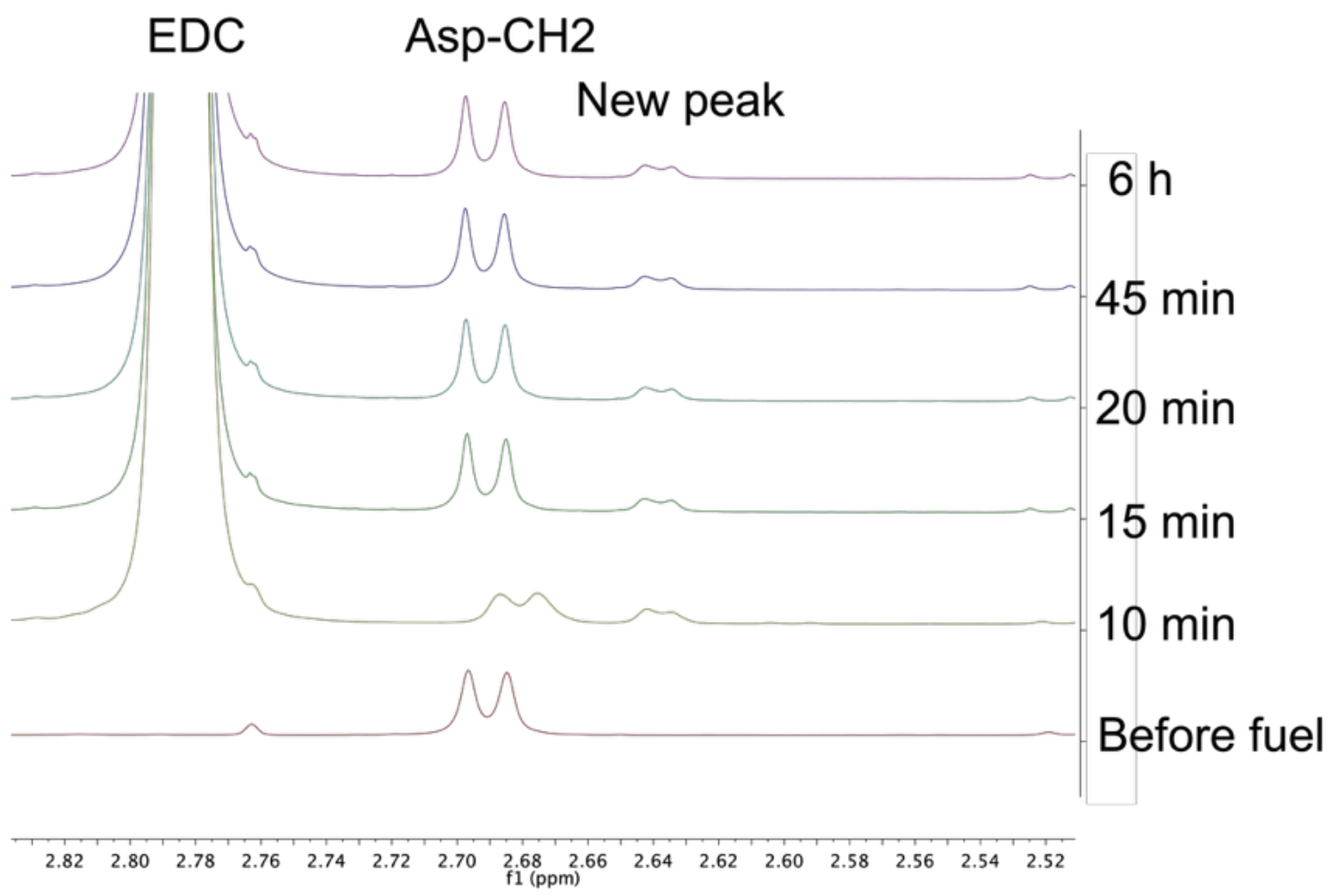

Fig. S11. Stacked 1H-NMR kinetics of W5 zoomed in on the aspartic acid CH2 peak. 6mM peptide was prepared in water (10% D2O) and fueled with 50mM EDC. Sample was measured on a Bruker EVO 500MHz NMR.

References

1. Tena-Solsona M*, et al.* Non-equilibrium dissipative supramolecular materials with a tunable lifetime. *Nature Communications* **8**, 15895 (2017).

2. Donau C*, et al.* Active coacervate droplets as a model for membraneless organelles and protocells. *Nature Communications* **11**, 5167 (2020).

3. Späth F*, et al.* Molecular Design of Chemically Fueled Peptide–Polyelectrolyte Coacervate-Based Assemblies. *Journal of the American Chemical Society* **143**, 4782-4789 (2021).
